# Genome-wide association studies in preterm birth: implications for the practicing obstetrician-gynaecologist

**DOI:** 10.1186/1471-2393-13-S1-S4

**Published:** 2013-01-31

**Authors:** Siobhan M Dolan, Inge Christiaens

**Affiliations:** 1 Department of Obstetrics & Gynecology and Women’s Health, Albert Einstein College of Medicine, Montefiore Medical Center, Bronx, NY, 10461, USA; 2 Department of Physiology, University of Alberta, Edmonton, Alberta, T6G 2S2, Canada; 3 Department of Obstetrics and Gynaecology, Betsi Cadwaladr University Health Board Wrexham Maelor Hospital, Wrexham, LL13 7TD, United Kingdom

## Abstract

Preterm birth has the highest mortality and morbidity of all pregnancy complications. The burden of preterm birth on public health worldwide is enormous, yet there are few effective means to prevent a preterm delivery. To date, much of its etiology is unexplained, but genetic predisposition is thought to play a major role. In the upcoming year, the international Preterm Birth Genome Project (PGP) consortium plans to publish a large genome wide association study in early preterm birth. Genome-wide association studies (GWAS) are designed to identify common genetic variants that influence health and disease. Despite the many challenges that are involved, GWAS can be an important discovery tool, revealing genetic variations that are associated with preterm birth. It is highly unlikely that findings of a GWAS can be directly translated into clinical practice in the short run. Nonetheless, it will help us to better understand the etiology of preterm birth and the GWAS results will generate new hypotheses for further research, thus enhancing our understanding of preterm birth and informing prevention efforts in the long run.

## Introduction

In the upcoming year, the Preterm Birth Genome Project (PGP), an international consortium of investigators examining the genetics of preterm birth [1], plans to publish results of a large genome-wide association study (GWAS) in preterm birth. The product of much time and effort, such research promises to deliver substantial health benefits for women and families. However, results of a GWAS do not instantly translate into clinical applications that will improve birth outcomes and ultimately maternal child health. The translation of gene discovery into improved health outcomes is a process [2]. This paper will provide a brief overview describing the design and interpretation of a GWAS and then explore the implications for the practicing obstetrician gynaecologist.

### Goal of a genome-wide association study

Genome-wide association studies are designed to identify common genetic variations that are associated with certain health outcomes or diseases. Unlike the search for highly penetrant disease-causing gene mutations such as those underlying sickle cell anaemia and Tay-Sachs disease, GWAS attempt to identify single nucleotide polymorphisms, also known as SNPs, that are not strictly disease causing but rather increase or decrease one’s risk of disease. Highly penetrant genetic disorders are caused by a single mutation or a few genetic variants at most, whereas complex or multifactorial diseases are influenced by many genetic variants as well as environmental factors. Thus a GWAS is inherently searching for genetic variation that does not fit the model of a rare mutation leading to disease; rather it is searching to identify common genetic variation that contributes to the risk for disease. Such an endeavour adds many layers of complexity in terms of both carrying out GWAS as well as interpreting the results.

Of the three billion base pairs of the haploid human genome, several million contain individual DNA sequence variants or alleles [3]. SNPs are the most common variations in the genome with an estimated 10 million SNPs occurring in the human population. A SNP is simply a single base pair substitution at a particular locus (location) and humans have approximately 1 SNP per 300 base pairs in their genome [4]. Very interestingly, human beings differ from one another in their genetic make-up only by 0.1%. However, in this 0.1% of the genome lie key differences that can determine a person’s susceptibility to diseases or health outcomes such as preterm birth. While pregnancy is not a disease, adverse birth outcomes such as preterm birth or stillbirth will be considered as disease states for the purposes of this paper.

The goal of a GWAS is to identify particular SNPs in the genome that are associated with increased risk for or protection from disease. Common variation is generally present in greater than one percent of the population [5]. As mentioned earlier, this variation is not highly penetrant nor directly causes disease, but rather confers susceptibility or protection. The benefits of a GWAS are that one does not need a hypothesis as to which genes or which pathways are involved in the causation of the disease; the approach is hypothesis free. This can prove extremely useful in challenging arenas such as preterm birth where the etiology and mechanism of the condition remain elusive [6]. But it also means that results are not final answers. A SNP identified in a GWAS may not be the actual SNP that is causing the disease in question but rather it may be near or in linkage disequilibrium (LD) with the SNP that leads to a functional change. LD is the non-random association of alleles at two or more loci and it describes a situation in which some combinations of alleles or genetic markers occur more or less frequently together in a population than would be expected.

The results of a GWAS provide a mechanism for generating further hypotheses to better understand biology, pathophysiology and disease causation. They provide a clue as to where to look amongst the more than three billion base pairs making up the genome to identify important genetic regions, genes, and potential mechanisms. Several publicly available databases exist that list all GWAS studies and their findings published to date (Table 1).

**Table 1:** GWAS Resources

Source	URL
Catalog of Genome Wide Association Studies National Human Genome Research Institute	http://www.genome.gov/26525384

GWAS Central – A Genotype-Phenotype Association Database	http://databib.org/repository/543

## Genotyping

DNA for a GWAS can be collected from multiple sources. These include maternal and/or fetal blood, saliva and blood spots. Commercially available high-throughput genotyping platforms (Affymetrix and Illumina) are used for the genotyping of genomic DNA. Currently, these so-called SNP arrays can genotype over 5 million SNPs at the same time. SNP assays rely on the biochemical principle that nucleotide bases bind to their complementary bases (adenine binds to thymine and cytosine binds to guanine) [7]. Fragmented single-stranded DNA is hybridized to arrays containing up to 1 million unique nucleotide probe sequences. Each probe is designed to bind to a target DNA. Subsequently, the intensity of the signal associated with each probe and its target after hybridization is measured. This signal intensity is dependent upon the amount of target DNA in the sample, and the affinity between target DNA and probe. Clustering algorithms are then used to infer SNP genotypes from the intensity of the allele-specific probes.

One important concept to consider is linkage disequilibrium or LD. As mentioned previously, this is the non-random association of alleles at two or more loci. Certain combinations of alleles or genetic markers occur more or less frequently together in a population than would be expected from a random formation of haplotypes (series of SNPs close together in the genome) from alleles based on their frequencies. Two SNPs that are in strong LD may therefore serve as proxies for one another: genotyping one of the SNPs gives nearly complete information regarding the genotype of the other SNP. This explains why an array that genotypes 5 million SNPs effectively actually assays a larger proportion of human genetic variation than represented on the array. For maximum advantage, the current arrays are specifically designed to detect SNPs that correlate with, or tag, a large number of other SNPs in the human genome. The work of the International HapMap Project largely facilitated this concept [8]. General differences in the LD patterns among different populations exist and this can influence the SNPs that are selected to be genotyped in a GWAS.

## Genetics of preterm birth

Preterm birth is defined as birth before 37 completed weeks of gestation (WHO guidelines) and its rate has risen alarmingly over the past twenty years. After peaking in 2006 at 12.8, the preterm birth rate in the United States was reported at 12.2% in 2009, the most recent year for which data are available [9]. Among African Americans, the preterm birth rate in 2009 was 17.5%. Other developed countries also have shown rising preterm birth rates in the past decade [10-12]. Possible explanations for these high rates include the increases in multiple births, older maternal age, elective caesarean sections before 37 weeks of gestation, and the use of assisted reproductive technologies such as in vitro fertilization. However, the rise in preterm birth can only be partially attributed to these factors [13].

The majority of preterm deliveries are idiopathic preterm births and preterm births due to preterm premature rupture of fetal membranes. In both cases, much of the etiology is unknown [14]. While many environmental contributors to preterm birth such as stress, smoking, and inflammation, are identified, a large body of research suggests that genetic predisposition plays an important role, as reviewed by Dolan *et al. *[15]. The leading risk factor for a preterm delivery is a prior pregnancy resulting in preterm birth. Next to cervical length measurement, this is currently the single best predictor of preterm birth in multiparous women. Twin studies support the role of genetic risk factors in preterm birth by estimating the heritability at 20 to 40 percent [16,17]. Furthermore, mothers who were born preterm themselves have an increased risk of delivering their babies preterm [18]. Evidence also shows there is a large racial disparity in the etiology of preterm birth. The association between ethnicity, especially African ancestry, and preterm birth persists even if corrected for other risk factors such as socio-economic status and access to prenatal care. While evidence does not suggest that preterm birth is inherited in a classic Mendelian autosomal recessive or autosomal dominant fashion, a predisposition to preterm birth clearly runs in families. Therefore, GWAS will likely be a useful tool to identify the genetic contribution to preterm birth that influences this heritability.

## Design of a GWAS for preterm birth

The most common design of a GWAS is a case-control study in which a large number of DNA samples from cases and controls are analyzed to look for SNPs that are associated with the disease or condition under study. A generally accepted sample size of 1000 cases and 1000 controls is likely adequate for many GWAS, however the required sample size depends on many factors including the prevalence of the disease and the effect size of the alleles. As in any case-control study, most important to the success of the study is selecting cases that meet a standard and consistent definition of the condition under study. This is a particularly challenging issue in preterm birth research as the phenotype of preterm birth can be quite complex to define. At its simplest definition, preterm birth is defined by the World Health Organization as birth before 37 weeks gestation. But beyond that, the definition becomes much more complex. Is late preterm birth, defined as 34 0/7 to 36 6/7 weeks, the same phenotype as very preterm birth at < 32 weeks? Is a preterm birth that follows chorioamnionitis and preterm premature rupture of the membranes the same phenotype as a preterm birth that follows preeclampsia and a placental abruption, even if they both occur at 28 weeks gestation? While challenging for preterm birth researchers, the goal is to identify as clear and consistent a phenotype as possible. For the purposes of many studies, spontaneous singleton preterm birth is the phenotype of interest and thus multiple gestations, fetuses with birth defects, and major maternal medical complications such as hypertension are excluded.

Selection of controls must be carried out such that controls come from the same population as the cases and they must be similar in every way to the cases other than the occurrence of the disease. This is generally accomplished by identifying term controls from the same labour and delivery floors where the cases are identified. Furthermore, it is important that as much demographic and environmental risk data are collected about the study subjects, so that they can be considered and controlled for in the final analyses.

## Challenges in interpretation and translation of findings

The use of genome wide analysis is a very powerful research tool. It can be an important first step in discovering the genetic variations that are associated with preterm birth. Analytic challenges in the interpretation of findings from GWAS however require technical and analytic expertise and software designed to carry out such analyses [19,20]. The fact that GWAS are looking to identify common genetic variation associated with small effect sizes requires large study samples (at least 1000 cases and 1000 controls), which often leads to consortia coming together to pool data. Working with large teams to assure consistent phenotyping, accurate genotyping, and standard protocols across the study sites can be challenging, as well.

There are many challenges to interpreting the findings of a GWAS. Significant findings need to be interpreted in the context of the million comparisons that occur in a GWAS. In order to handle the multiple comparisons, a *p-*value of less than 5 x 10 -8 is considered significant in a GWAS. This *p-*value threshold is for 1 million SNPs. It roughly approximates a *p-*value of less than 0.05 (equal to a 1 in 20 likelihood that the finding was due to chance alone) when doing one million comparisons. Mathematically, this can be calculated as 0.05 / 1,000,000 = 5 x 10 -8. This is known as a Bonferroni correction, a conservative statistical method to counteract the problem of type I error (false positives) in multiple comparisons.

Population stratification is another major challenge in interpreting GWAS. Population stratification captures the idea that the study identifies a variant (SNP) associated with a characteristic of a population that is associated with the disease, not the disease itself. The SNP of study may have significantly different allele frequencies in two different populations and the frequency of these populations may differ between the case and control group. This induces an association of the SNP with case-control status when it is actually only associated with the population differences between cases and controls. One way to assure that the association between the genetic variant and the disease outcome is actually robust, therefore, is to replicate the finding in a second study of subjects from a different racial / ethnic background. Many journals indeed require replication of findings before publishing GWAS. In addition, computational methods are also available to correct for population stratification in the analysis of a GWAS. Thus far, most GWAS are conducted in populations from European descent. It is important to select a strict and consistent phenotype, and therefore the choice of one particular population is often made. Whether the findings of many of these studies are generalizable to non-European populations is largely unknown.

In complex conditions like preterm birth that are known to be multifactorial, which means they are influenced by both genetic and environmental factors, GWAS will likely only tell part of the story. Demographic and environmental factors may be as important as genetic factors in the etiology of preterm birth. The question remains therefore, how can we best integrate environmental data such as smoking data and stress with the GWAS findings? And what if, as in the case of human height where 20 SNPs where found to explain only approximately three percent of the variation in human height [21], genetic variation is found to explain only a small amount of the variation in the risk of preterm birth? This is an area where continued research is needed. As Wang *et al.* illustrate, one model for using gene environment interactions is targeting environmental interventions such as smoking cessation programs to individuals with specific genetic variants [22].

Studying pregnancy adds another layer of complexity: there are two patients – mother and newborn – and three genomes to consider: the maternal, paternal and child’s. Integrating genome wide data across three individuals in order to identify the etiology of a complex birth outcome such as preterm birth is a challenge that continues to require new methodologies and continued research.

## Implications of GWAS for the clinician

We are hopeful that GWAS will identify regions of the genome associated with preterm birth. They can then serve as a useful tool for researchers to learn more about the basic pathophysiology of preterm birth and the pathways leading to preterm labor. Furthermore, as in the case of many complex disease conditions, there is the promise that risk prediction profiles could be identified in which a number of genetic variants in the aggregate can help assess risk [23]. While this is not a short-term proposition, it provides a roadmap for research to understand who is at risk for or protected against preterm birth.

GWAS will not lower rates of preterm birth in the short run. In fact, some critics of GWAS suggest that they have not provided as much information as was hoped. Critics suggest that the genetic variation underpinning many disease states will be found by identifying in a number of rare variants, not common variants, and thus inquiry via GWAS will not likely lead to useful information [19,24]. But, in the case of some conditions such as Alzheimer’s disease and breast cancer, genetic variants have been identified that will likely serve useful roles in the development of risk prediction algorithms and targeted treatment.

What is clear, however, is that GWAS in the area of preterm birth will add new information regarding genetic variation and risk to the research arena surrounding preterm birth. They will likely help us learn more about the fundamental biology of preterm birth and the biology of its causation, not its symptoms. GWAS will contribute to our ability to assess personalized risk for preterm birth and hopefully guide pharmacogenomic interventions. In an area such as preterm birth, where the public health burden is tremendous and the clinical interventions are scarce, GWAS will enhance scientific inquiry by providing new ideas and data in an area that is rich for discovery.

## Competing interests

The authors declare that they have no competing interests.

## Authors’ contributions

SD and IC contributed equally to the preparation of the manuscript.

## Declarations

This article has been published as part of *BMC Pregnancy and Childbirth* Volume 13 Supplement 1, 2013: Preterm Birth: Interdisciplinary Research from the Preterm Birth and Healthy Outcomes Team (PreHOT). The full contents of the supplement are available online at http://www.biomedcentral.com/bmcpregnancychildbirth/supplements/13/S1.

All of the publication fees will be funded by the Preterm Birth and Healthy Outcomes Team Interdisciplinary Team Grant (#200700595) from Alberta Innovates - Health Solutions, formerly the Alberta Heritage Foundation for Medical Research.
